# High salt diet increases the pressor response to stress in female, but not male ET_B_-receptor-deficient rats

**DOI:** 10.14814/phy2.12326

**Published:** 2015-03-22

**Authors:** Joshua S Speed, Gerard D'Angelo, Paul A Wach, Jennifer C Sullivan, Jennifer S Pollock, David M Pollock

**Affiliations:** Division of Nephrology, Department of Medicine, University of Alabama at BirminghamBirmingham, Alabama, USA

**Keywords:** Blood pressure, Endothelin-1, Stress

## Abstract

Acute stress in both rodents and humans causes a transient rise in blood pressure associated with an increase in plasma endothelin-1 (ET-1). High salt (HS) intake also increases ET-1 production, and interestingly, blunts the pressor response to acute air jet stress in rats. We previously reported that female rats lacking functional ET_B_ receptors everywhere except sympathetic nerves (ET_B_ def) had a greater degree of hypertension in response to a HS diet compared to their male counterparts when measured by the tail cuff method. However, we now report that salt-induced hypertension is not different between sexes when measured by telemetry. Therefore, additional experiments were designed to test the hypothesis that female ET_B_ def rats are more sensitive to acute stress when on a HS diet. The pressor response, measured by telemetry, to acute air jet stress was similar between male transgenic control (Tg control) and ET_B_ def rats following chronic HS intake. In contrast, female ET_B_ def rats had a significantly greater pressor response (about twofold higher) than female or male Tg control or male ET_B_ def rats maintained on HS, a finding that cannot be explained by increased vascular reactivity to ET-1 in female rats as we observed that male ET_B_ def rats had a greater pressor response to i.v. infusion of ET-1 compared to females. Furthermore, HS feeding exacerbated the pressor response to ET-1 in both male and female ET_B_ def rats. Given our previous studies demonstrating that the ET_A_ receptor functions to reduce the pressor response to acute stress, these findings further support a role for the ET receptor system in the pressor response to acute stress and that female rats have reduced ET_A_ receptor activity when on a HS diet compared to males.

## Introduction

In recent years, the importance of endothelin in salt-dependent hypertension has been clearly established. Furthermore, the frequency of hypertension and salt-sensitivity is lower in premenopausal women compared to men. Endothelin plays an important role in blood pressure regulation not only through ET_A_-receptor-dependent vasoconstriction and ET_B_-receptor-dependent vasodilation, but also by control of sodium reabsorption in the thick ascending limb and collecting duct of the kidney (Nakano and Pollock 2012; Speed and Pollock 2013). The lack of ET_B_ receptor function produced by genetic deletion or pharmacological inhibition results in salt-sensitive hypertension (Pollock and Pollock 2001; Speed et al. 2011a,b). Long-term blood pressure control by the ET_B_ receptor involves regulation of sodium reabsorption in the renal collecting duct and protection from ET_A_ receptor activity within the vascular system.

In addition to renal and vascular systems, it is clear that both ET_A_ and ET_B_ receptor systems participate in short-term blood pressure reactivity by modulating sympathetic nerve activity. These effects appear to reverse the traditional roles of ET_A_ and ET_B_ receptors, which are to function as prohypertensive and antihypertensive mediators, respectively (Hyndman and Pollock 2013; Speed and Pollock 2013). Dai and colleagues demonstrated an important role for ET_B_ receptors in stimulating sympathetic ganglia production of superoxide that contributes to mineralocorticoid hypertension (Dai et al. 2004). Our laboratory has also published a series of studies showing that the pressor response to acute environmental stress using the air jet model is normally limited by ET_A_ receptor activity and enhanced by ET_B_ receptor activity (D'Angelo et al. 2006, 2010). While the mechanisms are still unclear, systemic blockade of ET_A_ in rats potentiates the acute pressor response to stress in rats (D'Angelo et al. 2005, 2006). This effect appears to be mediated by ET_A_ dependent increases in plasma norepinephrine during stress and increased vascular sensitivity to phenylephrine (D'Angelo et al. 2006).

The ET_B_-receptor-deficient (ET_B_ def) rat is a model of human salt-sensitive hypertension. Normally, this genotype is lethal by 4 weeks of age due to the lack of a functional enteric nervous system, but Gariepy and colleagues rescued these rats by inserting a transgene that expresses a functional ET_B_ receptor in adrenergic tissues. These animals are deficient of functional ET_B_ receptors on vascular endothelium and renal tubular epithelium (Gariepy et al. 1996, 1998). However, they do display a pressor response to ET_B_ agonists demonstrating that ET_B_-dependent adrenergic tone is maintained (Pollock et al. 2000).

**Figure 1:**
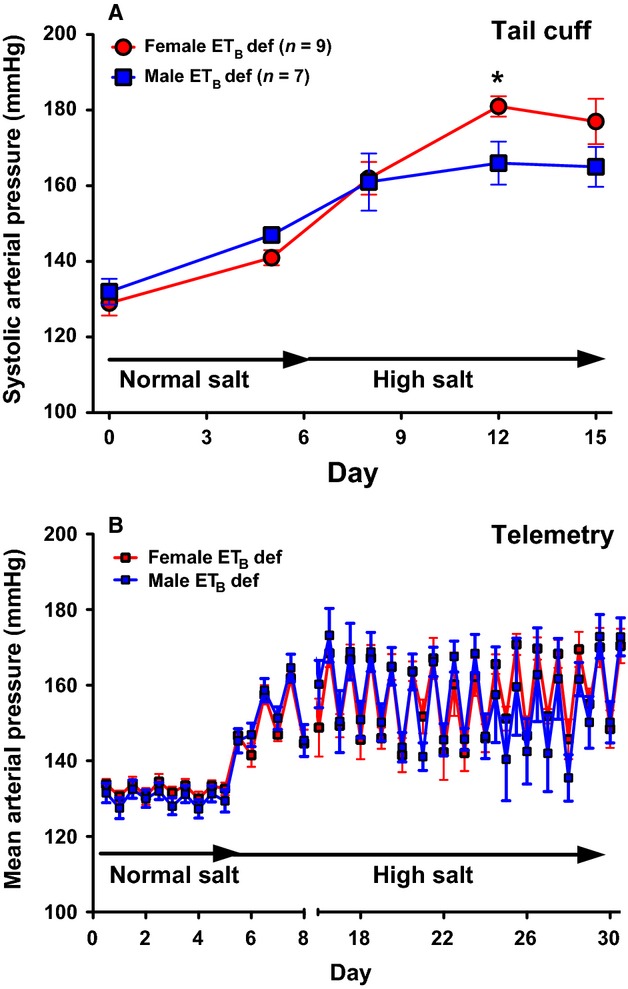
Comparison of blood pressure in male and female ET_B_ def rats. (A) Systolic blood pressure measured by tail cuff in male (*n* = 7) and female (*n* = 9) ET_B_ def rats before and after 2 weeks of HS feeding. (B) 12 h mean arterial pressure (MAP) in male (*n* = 7) and female (*n* = 9) ET_B_ def rats before and after 4 weeks of HS feeding. Data expressed as mean ± SE. *P < 0.05versus male.

Several years ago, we observed that female ET_B_ def rats became significantly more hypertensive than males when placed on a high salt diet (Fig.1A; Sullivan et al. 2006; Taylor et al. 2003). In these studies, blood pressure was measured by the tail cuff method. In more recent experiments using telemetric recording of blood pressure, we were surprised to see an identical elevation in blood pressure in both male and female ET_B_ def rats whether on a normal or HS diet (Fig.1B). These findings led us to hypothesize that female ET_B_ def rats have a greater pressor response to acute stress compared to males. Therefore, we conducted experiments to more directly compare the pressor response to acute environmental stress (air jet) in male and female ET_B_ def rats. Additional experiments were conducted to determine if vascular responsiveness to ET-1 could account for the sex-dependent differences as we know that acute stress increases circulating ET-1 concentrations (D'Angelo et al. 2002).

## Methods

Experiments used 9- to 12-week-old male and female littermate transgenic control (Tg control) and ET_B_ def transgenic rats from our local breeding colony. Prior to study, rats were maintained on a normal salt (NS) diet, that is, standard rodent chow containing 0.8% NaCl (Harlan Teklad; catalog # 8604) and tap water, *ad libitum*. A subgroup of male and female ET_B_ def rats were also placed on a high salt (HS) diet (8% NaCl; Harlan Teklad, TD.92034) for 3 weeks. All rats were housed in the animal care facility under conditions approved by the American Association for the Accreditation of Laboratory Animal Care. The Institutional Animal Care and Use Committee approved all procedures.

Blood pressure measurements were made using DSI PA-C40 transmitters (Data Sciences International, Duluth, MN) surgically implanted according to the manufacturer's specifications. In brief, rats were anesthetized with 2–3% isoflurane and the abdominal aorta was exposed by a midline incision, and briefly occluded. The transmitter catheter was inserted into the distal aorta and secured in place with VetBond tissue glue (3M corporation, St. Paul, MN). The transmitter body was secured in place along the incision line as it was closed with sutures. Staples were used to close the skin. Rats were allowed to recover for at least 1 week after surgery before being subjected to experimental protocols. Mean arterial pressure (MAP) and heart rate (HR) were recorded at 10-sec intervals every 10 min throughout the study.

### Acute air jet stress

The pressor response to acute air jet stress was determined in male and female Tg control and ET_B_ def rats as previously described (D'Angelo et al. 2010; Loria et al. 2010). Briefly, rats were acclimated to the stress protocol conditions by first placing them in a restraining cage for a period of 15–20 min each day over a period of several days. On the day of the air jet stress, blood pressure was monitored continuously by telemetry and rats subjected to air jet stress consisting of pulses (2 sec duration delivered every 10 sec for 3 min) of compressed air (15 lb/in^2^) aimed at the forehead from a 1/8” opening at the front of the tube. The total stress response was calculated as the sum of the change in MAP during the 3 min of air jet stress according to the equation: Σ((*P* − *P*_Base_) × 0.067)), where P refers to each MAP data point recorded during the delivery of air jet stress, *P*_Base_ is the average pressure during the 3 min just prior to the onset of the air pulses, and 0.067 is the 4 sec data collection interval in minutes. Data are expressed as the area under the curve (AUC; mmHg × min).

### Acute pressor response to ET-1

In a separate series of studies, the arterial blood pressure response to ET-1 and the ET_B_ agonist, sarafotoxin 6c (S6c), was determined in anesthetized male and female ET_B_ def rats (Inactin; 100 mg/kg, i.p.) using a previously published protocol (Pollock et al. 2000). Briefly, the right carotid artery and jugular vein were isolated and cannulated with PE-50 tubing for monitoring MAP and infusing ET-1 or S6c, respectively. After a 30-min equilibration period, animals were given chlorisondamine (5 mg/kg, i.v) to eliminate baroreceptor responses. Peptides were then administered at doses of 0.1, 0.3, and 1.0 nmol/kg, i.v., at 15-min intervals. All measurements were recorded using a PowerLab data acquisition system.

### Statistical analysis

All data are expressed as means ± SE. Statistical analyses of the total pressor response in male and female Tg control and ET_B_ def rats were made by two-way analysis of variance, followed by Newman-Keuls test for multiple comparisons. Pressor responses to ET-1 and S6c in anesthetized animals are reported as the area under the curve of the MAP tracing. Baseline was calculated by averaging the 4 min prior to first infusion of ET-1 or S6c. Significance was set at *P *< 0.05.

## Results

Since we previously observed that female ET_B_ def rats are more salt sensitive than males, as measured by tail cuff [Fig.1A adapted from Sullivan et al. (2006)], we next wanted to confirm this by telemetry. Baseline MAP was not different between male and female ET_B_ def rats on a NS diet when measured by telemetry (Fig.1B). In contrast to tail cuff data, there was also no difference in the blood pressure response to a HS diet, even after 4 weeks. This finding suggests that the sex difference observed by tail cuff may be due to stress induced by the tail cuff procedure.

**Figure 2:**
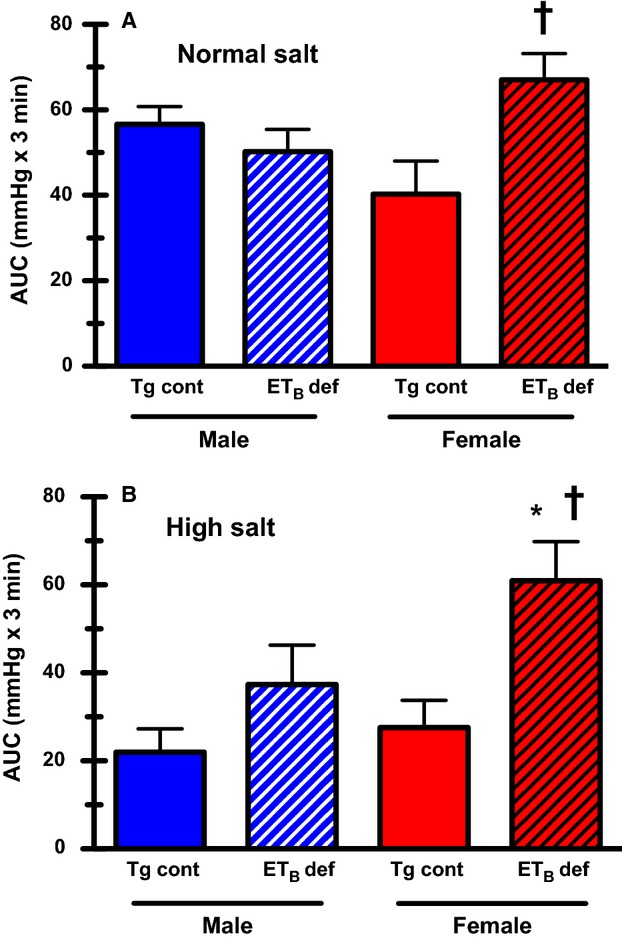
Total mean arterial pressure response to acute air jet stress expressed as area under the curve (AUC) in Tg control and ET_B_ def, male and female rats maintained on (A) NS diet (*n* = 5–6) and (B) HS diet (*n* = 5–6). Data expressed as mean ± SE; *P < 0.05 versus male Tg control; ^†^*P* < 0.05 versus female Tg control.

To determine if HS intake exacerbates stress-induced MAP increases in ET_B_ def females compared to males, we exposed rats to air jet stress, a well-established model of acute stress in rats (D'Angelo et al. 2010). The MAP response to air jet stress was similar between male Tg controls and ET_B_ def rats on NS chow (Fig.2); however, this response was significantly elevated in female ET_B_ def rats compared to female Tg controls. Interestingly, when placed on HS diet, resting MAP was not different between males and females, but the response to air jet stress was significantly greater in female ET_B_ def rats compared to female Tg controls. There was no significant difference in the pressor response to acute air jet stress between male Tg controls and ET_B_ def.

**Figure 3:**
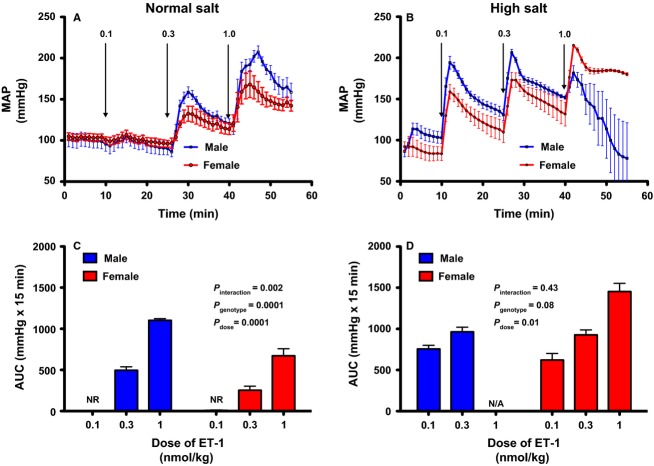
Comparison of MAP in response to intravenous bolus of increasing doses of ET-1 (0.1, 0.3, and 1.0 nmol/kg). Panels (A and C) represent MAP tracings from male and female (*n* = 3–5) ET_B_ def rats maintained on NS or HS diet, respectively, for 3 weeks. (B and D) represent the AUC after ET-1 infusion calculated from the baseline. Data expressed as means ± SE.

Because acute stress leads to a significant increase in plasma ET-1 (Treiber et al. 2002; D'Angelo et al. 2010), we hypothesized that the increased responsiveness to acute stress in female ET_B_ def rats maintained on HS was due to increased vascular sensitivity to ET-1. In anesthetized, ganglion-blocked rats maintained on a NS diet, i.v. infusion of ET-1 produced a dose-dependent increase in MAP in both male and female ET_B_ def rats (Fig.3A). The response to ET-1 was significantly greater in males versus females (Fig.3B). Interestingly, when maintained on chronic HS diet for 3 weeks, both males and females have a greater MAP response to bolus infusion of ET-1 (Fig.3B and D). Three of five male rats displayed cardiac arrhythmia, and died within minutes of the 1.0 nmol/kg bolus, so calculation of AUC was not relevant.

**Figure 4:**
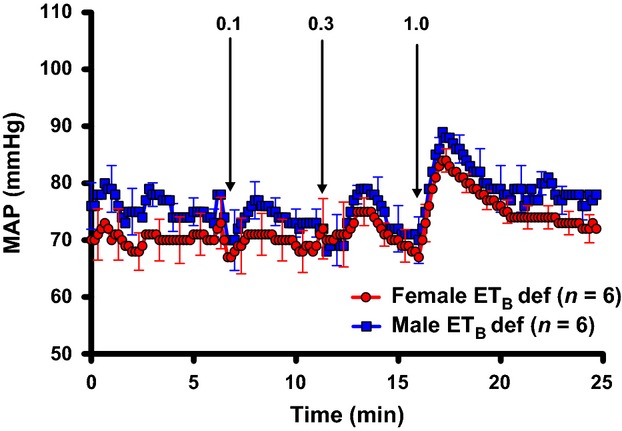
Comparison of MAP in response to increasing doses of S6c (ET_B_ agonist) in male and female ET_B_ def rats on NS diet. Data expressed as means ± SE.

ET_B_ def rats only express functional ET_B_ receptors on sympathetic nerves (Gariepy et al. 1998). Therefore, to determine if the increased sensitivity to stress in ET_B_ def female rats is due to an increased pressor response of ET_B_ receptors on the sympathetic nerves, we infused the ET_B_ agonist, S6c, similar to the previous experiment with ET-1. Although the pressor response to S6c was lower in ET_B_ def rats compared to previously published results in an intact strain, the acute pressor response to ET_B_ receptor activation was similar between male and female ET_B_ def rats (Fig.4).

## Discussion

One of the novel findings of this study is that female ET_B_ def rats have a greater blood pressure response to acute stress than males during chronic HS intake. This was observed in response to acute air jet stress as well as during tail cuff blood pressure monitoring in which the rats were placed in a restraining cage, a standard acute stressor (Irvine et al. 1997). In two independent studies using tail cuff methods, we previously reported that the systolic arterial pressure response to a HS diet in female ET_B_ def rats is elevated compared to males (Taylor et al. 2003; Sullivan et al. 2006). Typically, acute stress results in a dramatic, albeit short-lived, increase in blood pressure associated with an immediate increase in plasma ET-1 (Treiber et al. 2002; D'Angelo et al. 2010). Our laboratory has previously reported that the blood pressure response to air jet stress is blunted in both male Tg control and ET_B_ def rats maintained on a HS diet (D'Angelo et al. 2005). Inhibition of ET_A_ receptors restored the blood pressure response to the levels of normal salt fed Tg control rats, while exacerbating the stress-induced pressor response in ET_B_ def rats, providing evidence that activation of ET_A_ receptors blunts the blood pressure response to acute stress. In this study, we find a clear sex difference, such that the pressor response to air jet stress in ET_B_ def females is not blunted by HS diet, suggesting that intact females possibly rely more on the vasodilatory action of endothelial ET_B_ receptors to limit increases in blood pressure during acute stress.

It is well established that the body's response to acute stress includes a rapid increase in plasma ET-1 (Treiber et al. 2000, 2002). Plasma ET-1 concentrations are often difficult to interpret as the majority of endothelial-derived ET-1 is released toward the basal side of the cell and both ET_A_ and ET_B_ receptors bind ET-1 irreversibly (Speed and Pollock 2013). Therefore, plasma levels do not always reflect production, but can be an indicator of clearance. To complicate matters further, mice lacking ET-1 production have reduced (Kisanuki et al. 2010), but still measurable ET-1 levels, yet the origin of this ET-1 is not clear. Although we did not measure plasma ET-1 in this study, it has been shown in humans that males have a greater increase in plasma ET-1 compared to females (Treiber et al. 2002). There are numerous potential explanations for why female ET_B_ def rats have a greater blood pressure response to acute stress than males when placed on HS. However, we first considered that the increased stress response was due to greater vascular sensitivity to ET-1 causing a greater increase in pressure in female versus male ET_B_ def rats. Interestingly, on a NS diet, male ET_B_ def rats had a greater blood pressure response to i.v. infusion of ET-1 than females at 0.3 nmol/kg and 1.0 nmol/kg (Fig.3B). The response to ET-1 in rats on a HS diet was clearly increased compared to NS diet animals of either sex, but again, ET-1 was less potent in female rats. Thus, our original hypothesis of greater vascular reactivity in female rats was not supported by our observations. In fact, three of the five male rats on a HS diet did not survive the highest dose of ET-1 whereas all the females survived. We have previously reported that male rats with intact ET_B_ receptor function in the endothelium can tolerate these doses of ET-1 (Pollock et al. 2000). It is clear, therefore, that HS intake increases the sensitivity to ET_A_ receptor activation in rats without an endothelial ET_B_ receptor consistent with a role for the ET_B_ receptor to protect against ET_A_ receptor activation, especially on a HS diet (Kohan et al. 2011a,b).

Our original hypothesis that increased vascular sensitivity to ET-1 in female ET_B_ def rats mediates the increased pressor response during high salt intake was clearly not supported; however, numerous other mechanisms could explain our results. For example, female rats have been shown to have higher blood volume than male rats and so a given level of sympathetic stimulus could cause a larger increase in MAP especially when on a high salt diet (Probst et al. 2006). Other possibilities include differential changes in circulating hormones and electrolytes produced by high salt intake that could modulate an acute stress response.

As seen in Fig.4, i.v. infusion of the ET_B_ agonist, S6c, still elicits a blood pressure response in ET_B_ def animals, even though there are no functional receptors on the vasculature. Therefore, we presume this response must be mediated by activation of the transgene in adrenergic tissues as described previously (Gariepy et al. 1998). Prior work in our laboratory suggests that stimulation of ET_B_ receptors on the sympathetic nerves leads to vasoconstriction (Pollock et al. 2000). We hypothesize that neural ET_B_ receptor stimulation results in *α*-adrenergic activation as a means of increasing vascular resistance. Here, we show that activation of the neuronal ET_B_ receptors elicits a similar blood pressure response in male and female ET_B_ def animals. Thus, it appears that sex differences in the acute response to stress are mediated primarily through ET_A-_receptor-dependent mechanisms.

One interesting finding of this study is that chronic HS feeding enhances the pressor response to exogenous ET-1 in ET_B_ def rats (Fig.3). The mechanisms responsible for the increased pressor response have yet to be determined; however, within the kidney, HS diet has been shown to alter ET-1 receptor binding. Specifically, Schneider and colleagues have shown that a high salt diet increases ET_B_ receptor binding and functional activity within the renal vasculature of male Sprague–Dawley rats (Schneider et al. 2007). In the renal inner medulla where ET_B_ receptor expression is primarily within the collecting duct, rats and mice maintained on HS diet have a reduction in ET_A_ receptor binding while maintaining ET_B_ receptor binding compared to animals on NS (unpublished observations). In normal Sprague–Dawley rats, chronic ET_B_ receptor blockade produces a greater degree of hypertension in male, compared to female rats and chronic angiotensin II induced hypertension reduces ET_B_ receptor function to a greater extent in male versus female rats (Kittikulsuth et al. 2013). These findings support the idea that ET_B_ receptor signaling is functionally preserved in female, compared to male rats. Furthermore, studies in ovariectomized rats suggest that estrogen may have an important role in maintaining functionality of the ET_B_ receptor (Nakano and Pollock 2009).

The sex difference observed in ET-1 receptor signaling is certainly not limited to the vasculature or neuronal tissue. Within the kidney, ET-1 activates ET_B_ receptors on the collecting duct to inhibit Na^+^ transport; however, females also have an ET_A-_receptor-dependent pathway to inhibit transport by the collecting duct that is absent in males (Nakano et al. 2008; Nakano and Pollock 2009). Furthermore, infusion of ET-1 into the renal artery or the medullary interstitium reduces medullary blood flow in male rats, but not females, consistent with increased vascular responsiveness observed in this study. Interestingly, it has also been reported that female and male rats have different ET-1-receptor-binding profiles in the inner medulla, in that females have much less ET_A_ receptor binding than males (Jin et al. 2013). Therefore, it is not surprising that ET-1 mediated effects are through distinct pathways in male versus female rats. More work needs be performed to fully elucidate these mechanisms and to determine how sex steroids affect ET-1 receptor expression and signaling.

## Conclusion and Perspectives

One important perspective gained from this study highlights the usefulness of measuring blood pressure in rodents by telemetry rather than tail cuff, because stress, whether chronic or acute, has dramatic effects on blood pressure and cardiovascular health (D'Angelo et al. 2010; Loria et al. 2010). Here, our findings suggest that ET-1 plays an important role in limiting the pressor response to acute stress through activation of the ET_A_ receptor consistent with our previous results (D'Angelo et al. 2005; Loria et al. 2010), and this limiting role is attenuated in female rats lacking endothelial ET_B_ receptors. This sex difference is more profound when rats are maintained on a HS diet. Clearly more work is needed to discern the physiological role of the ET system within the peripheral nervous system. Understanding specific mechanisms by which stress influences blood pressure between the sexes will be instrumental for the development of more individualized treatment plans for patients in the future.
